# Redundancy-Based Motion Planning with Task Constraints for Robot Manipulators

**DOI:** 10.3390/s25061900

**Published:** 2025-03-19

**Authors:** Yi Zhang, Hongguang Wang

**Affiliations:** 1 State Key Laboratory of Robotics, Shenyang Institute of Automation, Chinese Academy of Sciences, Chuangxin Road 135, Shenyang 110016, China; zhangyi1@sia.cn; 2 University of Chinese Academy of Sciences, Yuquan Road 19, Beijing 100049, China

**Keywords:** motion planning, task constraint, redundant manipulator

## Abstract

Finding realistic motions for redundant manipulators is essential for complex jobs such as home care and industrial assembly. Motion planning is complex when a task requires standing upright or moving through restricted spaces. This work provides an effective motion-planning strategy for 7-DOF manipulators that improves connections via redundancy. The analytic Cartesian-space-to-joint-space kinematic mapping models for 7-DOF redundant manipulators with diverse configurations are constructed first, and the feasible nodes are determined by sampling the Cartesian space without barriers to satisfy the task requirements. Each Cartesian-space sampling node can provide numerous feasible joint-space nodes because of the redundancy of the robot manipulators. To remove additional valid nodes from a singular position, joint configurations with the same end-effector position orientation are modified iteratively. Finally, we find the nearest nodes in the joint-space constraint manifold and build collision-free smooth pathways. The task constraint levels were varied for a 7-DOF manipulator in simulations and experiments. The proposed planner finds more viable nodes at the same end-position attitude than one-to-one projection. It does not require numerical iterations and achieves high planning efficiency and a high motion-planning success rate.

## 1. Introduction

**Figure 1:**
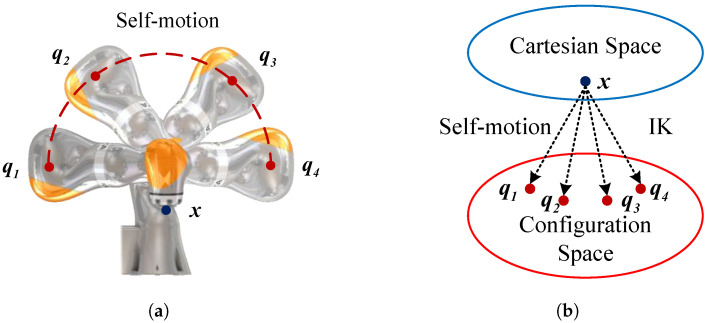
7-DOF redundant manipulator and its self-motion. (**a**) The Cartesian-space end-effector pose has a one-to-many relationship with joint-space nodes. (**b**) The 7-DOF manipulator kinematics are redundant, resulting in infinite kinematic inverse solutions.

Advances in robotics enable the widespread use of multi-degree-of-freedom robots in daily life, industrial production, and practical applications. The generic 7-DOF redundant manipulator and the anthropomorphic arm are the main types of multi-degree-of-freedom robots [1]. While keeping the end-effector position fixed, the elbow joint can rotate around the joint shoulder–wrist axis. However, manufacturing and actuation limitations cause differences in the joint axes of 7-DOF manipulators, especially at the shoulder and wrist joints. Robot manipulators with offsets are more flexible and work in a specific direction. Examples of 7-DOF redundant manipulators with zero offsets include humanoid robot manipulators with S-R-S configurations [2]. Figure 1 shows that the 7-DOF robot manipulator has unlimited inverse kinematic solutions due to kinematic redundancy. This lets the end-effector follow the same path while the joints move differently. The manipulator’s redundant kinematics reduce obstacles, prevent unique configurations, ensure fault tolerance, and optimize arm dynamics [3].

Sample-based motion planning is the primary method for high-dimensional, multi-degree-of-freedom robot manipulator systems, including anthropomorphic arms. These methods randomly sample the robot’s configuration space and link the start and goal settings to find a path solution to create collision-free graphs. The manipulator’s end-effector moves along the intended Cartesian trajectory through collision-free joint motions. Robot manipulators that perform realistic operations are gaining attention. Examples include opening doors, handling trays or cups of water, assembling industrial products, and machining. The end-effector must be restricted to the motion path, and the target joint configuration must be finalized during motion planning. These task limits complicate robot manipulator motion planning. Sample-based motion-planning methods under task restrictions extensively use sampling locations on the constrained manifold [4]. The degrees of freedom of a robot manipulator and the activity restrictions complicate motion planning [5]. Under this constraint, the robot’s end-effector positions and orientations are usually nonlinearly related, making inverse kinematics resolution difficult. Second, the sampling method randomly samples its configuration space, making it unlikely that points of a robot manipulator with a limited manifold will be sampled [6]. In particular, direct sampling of the configuration under constraints is impossible when the constrained manifold has zero volume in the configuration space [4].

Recently, many sampling-based motion-planning methods have been presented to address these concerns [7,8]. Relaxation methods increase the permissible tolerance of constraints to expand the subset of manifolds that comply with the limitations and solve the constraint problem when no viable configuration exists under strict restrictions. Bialkowski used this method to sample manifolds uniformly, simplifying the constant factor and improving motion planning in challenging domains [9]. Massimo solved collision-free motion-planning problems with relaxed soft constraints on manifolds, which are suitable for situations without strict restrictions [10]. This method may be better for sampling narrow channels depending on the robot controller performance. Its numerical motion-planning precision decreases as limitations are intentionally eased.

Jacobi matrix projection and numerical iteration are often used to plan robot manipulator movements while respecting location constraints [11]. Gradient descent via a projection operator maps a precise configuration onto a constrained manifold and uses iterative optimization to solve the constraint equations. The Jacobi matrix projection operator is often used. Berenson created a confined bidirectional rapidly exploring random tree method [12]. Over time, the sample projection finds an appropriate constraint function configuration. For simplicity of design, the sampling method projection operator can be cyclically projected under numerous constraint systems via more advanced solvers [13]. The numerical iterations are time-consuming since this method cannot avoid joint limits and singularities and must solve complex systems of equations defined by constraints [14]. Not all restriction Jacobians are invertible. If the constraint function’s Jacobian matrix has a complete rank, the tangent space can approximate the flow shape, and local perturbation can yield configurations that roughly satisfy the constraints. Tangent-space sampling improves accuracy with more residual dimensions in the limited manifold approximation, but the processing time increases. Yakey projectively rectified proximal samples via tangent space projection [15]. The limited ratio is expensive to calculate via the tangent space technique and is inapplicable at singularities and manifold regions of large curvature. To avoid recomputation, the atlas-based technique maintains the tangent space [16]. This method uses segmented linear approximation to cover the constrained manifold’s tangent space. Kim proposed the tangent bundle rapidly exploring random tree (TB-RRT), which interpolates only within the tangent space, improving computing performance; however, the samples are not uniform [17]. Atlas-based representation combines complexity with computing efficiency. An efficient and precise data structure is needed for this method, and diminishing returns on the residual dimension of the constrained flow in the ambient configuration space make the flow shape approximation method computationally inefficient [18].

Several studies have been conducted on direct task-constrained environments and on the projection approach and its modifications. Xia presented an FMT planner [19] to study bitmap space, which uses self-motion manifolds to sample an unobstructed Cartesian space bidirectionally. This technique works only for S-R-S anthropomorphic arms despite integrating inverse kinematics with motion planning. Wang’s direct projection planner [20] used rapidly exploring random trees. The principal arm and wrist joints are independently regulated, separating the robot manipulator’s end-effector position from its orientation. Compliant configurations are joined on the limited manifold to provide smooth, collision-free configurations. This strategy only applies to anthropomorphic arms and limits robot manipulator redundancy. Jang’s motion-planning method with closed-chain constraints randomly chooses the goal and iteratively improves the start and target nodes through inverse kinematics to generate feasible nodes [21]. A numerical iteration method was used to solve the inverse kinematics problem. However, it is time-consuming and delivers only one pair of Cartesian-to-joint-space solutions per iteration, reducing the robot manipulator’s mobility. This method modifies the start and target points to improve graph connections. However, it does not work when the nodes’ positions are predetermined. Many previous methods are either not replicable and are limited to specific robot manipulator configurations or cannot fully leverage the redundancy of the 7-DOF robot manipulator to improve joint mobility.

Optimization-based motion-planning techniques are broad, even if the prior subjects were all sampling-based. Computing the hybrid optimization problem the specified optimization strategy creates is frequently challenging [22]. However, its sensitivity to the setting conditions could cause the plan to fail [23]. Recent developments in integrating data-driven techniques with trajectory optimization have demonstrated the capacity to forecast the trajectories produced when used with model-based trajectory optimization techniques [24,25]. These learning approaches have great potential to enhance the effectiveness and versatility of the motion-planning procedure. Both control and state are regarded as choice variables in this simple transcription, which makes it simple to describe complex state constraints. A general-purpose NLP solver can be used to find the best answer. By learning a neural network, Sharma converts natural English sentences into a cost function transformation that is then utilized to optimize the motion trajectory [26]. In reality, though, optimization-based approaches frequently become stuck in local minima, particularly in intricate, unstructured settings. Our study can also benefit from other application domain-based motion-planning techniques. For example, some agricultural research uses motion-planning techniques for robot manipulators [27,28,29].

RBRRT is a redundancy-based, rapid-exploration, random-tree motion-planning method with task constraints for a 7-DOF redundant manipulator. Our motion-planning technique incorporates robot kinematics and motion planning while addressing the joint redundancy of the 7-DOF robot manipulator. Our procedure improves the efficiency of motion planning and reduces its duration.

- Samples in Cartesian space are used to calculate configurations that match constraints directly, and the sampling dimension decreases as the number of task restrictions increases. The robot manipulator kinematics are integrated with motion planning.
- Analytical inverse kinematics derive joint configurations that match constraints, reducing the iteration time of typical iterative approaches.
- Redundant inverse kinematic self-motion generates many feasible joint position nodes, avoiding joint constraints and singularities and improving sampling points and graph connections.

**Figure 2:**
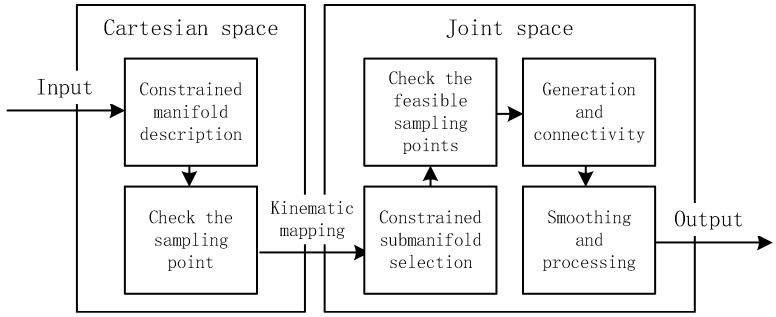
Flowchart of the proposed motion-planning method.

The provided method can solve the inverse kinematics of a general 7-DOF redundant manipulator with offsets and an anthropomorphic arm with an S-R-S structure. The technique was evaluated through simulations and tests on a 7-DOF rotary robot manipulator with offsets. A redundant manipulator arm with degrees of freedom, offsets, and limits has many advantages over standard arms. Switching and transitioning within the joint space submanifold is easy with RBRRT. Figure 2 displays the overall flowchart of the proposed motion-planning method.

The paper is organized as follows: Section 2 describes pre-motion-planning preparations. Section 3 details the proposed motion-planning method. Section 4 describes the experimental platform simulation environment and compares the proposed planner with conventional methods. Section 5 discusses the conclusions and future work.

## 2. Preliminaries

### 2.1. Constraint Expression

The primary strategy in motion planning is to transform the problem of orchestrating a three-dimensional, multi-degree-of-freedom robotic system into determining a singular point that represents the robot in a high-dimensional space. We characterize a robot with an intricate configuration as a singular point within the configuration space *Q*. The motion-planning problem involves finding a continuous path $\tau :\left[0,1\right]\to Q_{free}$ from the starting configuration $q_{start}=\tau \left(0\right)$ to the goal configuration $q_{goal}=\tau \left(1\right)$ in the free space $Q_{free}$, where $q_{start},\;q_{goal}\subseteq Q_{free}$.

When the robot performs a task under limits, its movements are limited relative to the free region. With *m* degrees of freedom reduced by the restriction (n>m>0), the constraint function $F:Q\to R^{n-m}$ is defined. When *q* meets all requirements, F(q)=0. Positional task restrictions limit the robot’s execution component, the end-effector. Positional and orientational limitations on the end-effector are commonly represented by the world coordinate system’s 6×2 boundary matrix $B^{\omega }$:

$$B^{\omega }=\left[\begin{array}{cc} x_{min} & x_{max}\\ y_{min} & y_{max}\\ z_{min} & z_{max}\\ \psi_{min} & \psi_{max}\\ \theta_{min} & \theta_{max}\\ \phi_{min} & \phi_{max} \end{array}\right]$$

The first three rows represent translational components that restrict movement along the *x*-, *y*-, and *z*-axes, and the last three rows denote rotational components that constrain rotation around these axes [30]. The end-effector constraints can be denoted as $F\left(q\right)=B^{\omega }$ [31].

### 2.2. Redundant Inverse Kinematics

**Figure 3:**
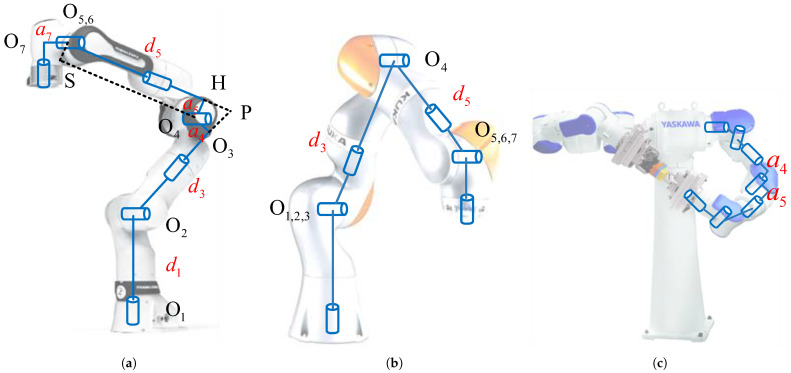
Kinematic model of the 7-DOF redundant manipulator. (**a**) Robot manipulator with offset (Franka Emika Panda), (**b**) anthropomorphic manipulator (Kuka iiwa), (**c**) robot manipulator with a similar configuration to Franka (Yaskawa SDA5D).

This section presents the generic analytical inverse kinematics of a 7-DOF manipulator for converting Cartesian-space nodes that meet the requirements into joint-space nodes that adhere to the constraints. This study focuses exclusively on a 7-DOF redundant manipulator comprising solely revolute joints, the predominant configuration available on the market. Figure 3 illustrates the kinematic model of a 7-DOF redundant manipulator. Using the Franka Emika Panda as an example, it is infeasible to compute the inverse kinematics via the conventional redundant arm angle approach with an S-R-S configuration with joint offsets [1]. Consequently, we designate $q_{7}$ as the redundant joint angle, and the remaining six joints serve as the primary working joint angles for calculating the kinematic chain from the terminal end [32]. The associated joint angles are determined via the analytical inverse kinematics method. $d_{1}$, $d_{3}$, and $d_{5}$ represent the lengths of the shoulder, upper arm, and forearm, respectively, and $a_{4}$, $a_{5}$, and $a_{6}$ denote the offsets. In this section, qi represents the joint angle value, and Rij=xijyijzij denotes the orientation of coordinate system *i* relative to coordinate system *j*.

This is a full description of the procedure, which includes the following steps:

- Seven joint variables in the 7-DOF redundant manipulator correspond to six spatial variables in Cartesian space. Therefore, $q_{7}$ is selected as the redundant variable. The connection of the shoulder, elbow, and wrist points creates a triangle $▵O_{2}O_{4}O_{6}$.
  (1) $$\begin{array}{cc} \bar{O_{2}O_{4}} & =\sqrt{d_{3}^{2}+a_{4}^{2}}\\ \bar{O_{4}O_{6}} & =\sqrt{d_{5}^{2}+a_{5}^{2}} \end{array}$$
- At the same position, the triangle $▵O_{2}O_{4}O_{6}$ is symmetric about the line $O_{2}O_{6}$.
  $$p_{7}=p_{EE}-(d_{F}+d_{EE})z_{EE}$$
  The robot manipulator has two equivalent states of elbow down and elbow up, and the following equation calculates the two sets of elbow joint angles $q_{4}$.
  (2) $$q_{4}=\left\{\begin{array}{c} ∠O_{2}O_{4}O_{3}+∠HO_{4}O_{6}+∠O_{2}O_{4}O_{6}-2\pi \\ ∠O_{2}O_{4}O_{6}-∠O_{2}O_{4}O_{3}-∠HO_{4}O_{6} \end{array}\right.$$
  where $∠O_{2}O_{4}O_{3}=\arctan\frac{d_{3}}{a_{4}}$
  $∠HO_{4}O_{6}=\arctan\frac{d_{3}}{a_{5}}$
  $∠O_{2}O_{4}O_{6}=\arccos\frac{{\bar{O_{2}O_{4}}}^{2}+{\bar{O_{4}O_{6}}}^{2}-{\bar{O_{2}O_{6}}}^{2}}{2\cdot \bar{O_{2}O_{4}}\cdot \bar{O_{4}O_{6}}}$.
- For each elbow angle $q_{4}$, there are two equivalent states of wrist angle $q_{6}$: not flipped and flipped.
  $$∠O_{2}O_{4}H=∠O_{2}O_{4}O_{6}+∠HO_{6}O_{4}$$
  (3) $$q_{6}=\left\{\begin{array}{c} \pi -\psi_{6}-\phi_{6}+2k\pi \\ \psi_{6}-\phi_{6}+2k\pi \end{array}\right.$$
  where ϕ6=atan2(y266,x266).
  ψ6=arcsinO2O6¯·cos∠O2O6Hx2626+y2626
- For each elbow–wrist combination, there are two possibilities for $\left(q_{1},q_{2}\right)$ pairs; right- and left-handed kinematic configurations.
  $$∠PO_{2}O_{6}=∠O_{3}O_{2}O_{4}+∠O_{4}O_{2}O_{6}$$
  (4) $$\begin{array}{c} \left\{\begin{array}{c} q_{1}=\mathrm{atan}2\left(y_{2P},x_{2P}\right)\\ q_{2}=\arccos\frac{z_{2P}}{\bar{O_{2}P}} \end{array}\right.\\ \left\{\begin{array}{c} q_{1}=\mathrm{atan}2\left(-y_{2P},-x_{2P}\right)\\ q_{2}=-\arccos\frac{z_{2P}}{\bar{O_{2}P}} \end{array}\right. \end{array}$$
- A singularity occurs when $q_{2}=0$ (pointing upward). The atan2 function fails because $x_{2P}$ and $y_{2P}$ may be zero simultaneously. In this state, joints 1 and 3 are coaxial, and there are infinite solutions for $q_{1}$ and $q_{3}$. Joints 1 and 3 can rotate freely about the *z*-axis independently of the other joints. A default value for $q_{1}$ must be predefined, typically set to the value of the previously feasible position. The solutions for joint angles $q_{3}$ and $q_{5}$ can be obtained using the values $q_{1}$, $q_{2}$, $q_{4}$, and $q_{6}$ obtained earlier.
  (5) q3=atan2(zx32,xx32)
  (6) q5=−atan2(y5S5,X5S5)
  *S* is the projection of $O_{4}$ on the $X_{5}-Y_{5}$ plane.

**Figure 4:**
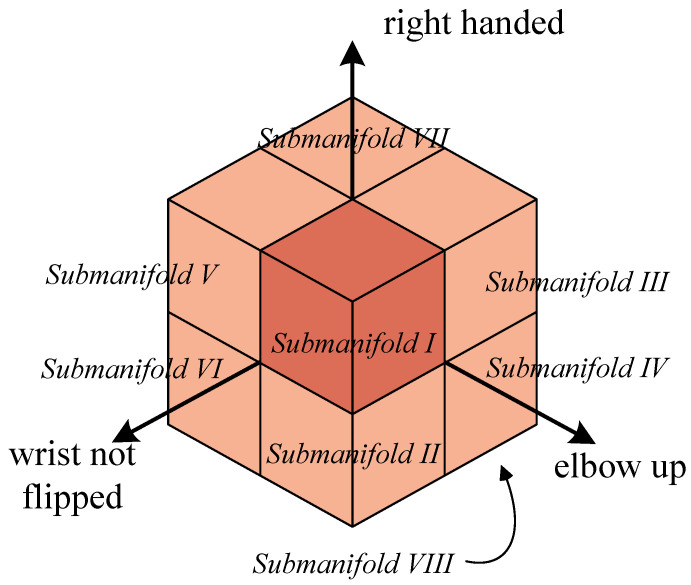
Self-motion manifolds in joint space, divided into eight submanifolds according to the joint motion range and submanifold definition. For Franka Emika Panda, the submanifolds are defined by elbow down: $q_{4}<-26.76{}^{\circ}$, wrist not flipped: $\vec{O_{2}O_{6}}\cdot x_{5}<0$, and right handed: $q_{2}>0$; the default submanifold is I (right–up–no-flip).

On the basis of the range of motion of the joint and the definition of the submanifold, it is divided into eight submanifolds, as shown in Figure 4. In each submanifold, $q_{7}$ is used as the unique variable so that the Cartesian-space and joint-space inverse kinematics solutions are in one-to-one correspondence. The combination right–up–no-flip is chosen by default. Some parameters are factors of the kinematic equations, and their values are easily found from their geometric relationships.

The particular case of the S-R-S structure with offset 0 is shown in Figure 3b. For anthropomorphic manipulators, $a_{4}=a_{5}=a_{7}=0$. Other 7-DOF redundant manipulators with similar configurations can also apply the method, as shown in Figure 3c. The Yaskawa SDA5D can also be directly applied to the above kinematic analysis, which only requires $a_{7}=0$.

## 3. Proposed Algorithm

The RBRRT algorithm is proposed to perform motion planning for a 7-DOF redundant manipulator to find collision-free paths on constrained manifolds. The method combines the inverse kinematic mapping relation above for the redundant manipulator and the rapid-exploration random tree (RRT) algorithm. The technique first uses RRT on a Cartesian space-constrained manifold, and each acquired node obtains multiple feasible joint-space sampling points via the mapping function Map for $q_{7}$. The nodes are extended and connected via the RRT single-query algorithm to ensure motion smoothness for the end-effector and joint trajectories while avoiding joint motion constraints and singularities. In this section, $q_{i}$ denotes a joint-space sampling point with seven dimensions, and $x_{i}$ denotes a node in Cartesian space with six dimensions.

Algorithm 1 illustrates the mapping function Map that converts Cartesian space to joint space for RBRRT. It integrates inverse kinematics and a choice of submanifolds of the joint space under constraints. After selecting $q_{7}$ as a redundant variable, the remaining joint angles $q_{1}$ through $q_{6}$ can be determined analytically. Consequently, eight sets of analytical inverse kinematics solutions can be derived when the end-effector pose and redundant angle variables are specified. According to the decision criteria, we categorize these eight solution groups into distinct submanifolds. The current posture can be determined from the previous joint angle $q_{a}$, which defines the unique submanifold of the joint space where the solution resides, under constraints. The transition points of the submanifolds are examined concerning three boundary conditions: elbow orientation, wrist positioning, and left- or right-handed kinematic configurations ($q_{4}=-26.76{}^{\circ}$; $\vec{O_{2}O_{6}}\cdot x_{5}=0$; $q_{2}=0$). Identifying the precise submanifold and ensuring the consistency of the inverse kinematics computational function enables the robot manipulator’s posture to be determined during motion planning and execution, preventing unexpected complications arising from submanifold transitions that result in significant motions.

The distribution is examined as follows. Initially, the position and orientation of the Cartesian-space robot manipulators are established on the basis of the actual joint angle from the preceding point (lines 1–4). The elbow connection is established on the basis of the value of the fourth joint angle $q_{4}$ concerning the preceding joint angle $q_{a}$ as a criterion for determination. When the elbow is positioned downward, the Boolean value of the current state $A_{cur}$ is assigned True; otherwise, $A_{cur}$ is assigned False. The formula for $q_{4}$ is presented in (2) (lines 5–8). Subsequently, it is determined whether the wrist of the robot manipulator is deflected; when $\vec{O_{2}O_{6}}\cdot x_{5}\left(q_{a}\right)\leq 0$, the wrist remains unflipped, and the state $B_{cur}$ takes the value True; otherwise, it takes the value False. $q_{6}$ is computed via Formula (3) (lines 9–12). Ultimately, left- and right-handed kinematic designs are evaluated. The singularity issue of $q_{2}$ must be addressed. $q_{2}$ equals zero when the vector $\bar{O_{2}P}$ is oriented upward. $q_{1}$ and $q_{3}$ have an infinite number of solutions, and joints 1 and 3 can be rotated indefinitely around the *z*-axis without altering the position of the robot manipulator. Therefore, when $q_{2}\geq \left|d\right|$, $q_{2}$ is positive away from the singularity for a right-handed configuration with $C_{cur}$ True, and the opposite holds for a left-handed configuration with $C_{cur}$ False. The equations for $q_{1}$ and $q_{2}$ are defined by (4) (lines 13–20). When $q_{2}<\left|d\right|$ and $q_{2}\geq 0$, $q_{2}$ approaches the singularity. For submanifolds of the joint space under constraints at the intersection of two submanifolds, *d* is the maximum step size of $q_{2}$. To facilitate seamless transition between the anterior and posterior points of the two submanifolds, $q_{2}=0$ and $q_{1}=q_{1}\left(q_{a}\right)$ (lines 16–20) are set. After determining all configurations at the shoulder, elbow, and wrist, namely, the associated submanifolds within the joint space, we calculate the values of the joint angles $q_{3}$ and $q_{5}$ via Equations (5) and (6) (lines 21). After multiple iterations of numerical calculations, the joint angles must remain within the permissible limits of the joints (lines 22 and 23). The sole viable joint angle $q_{1}$ is produced with $A_{cur}$, $B_{cur}$, and $C_{cur}$ to establish the submanifold of the joint space under constraints (lines 24 and 25).
**Algorithm 1:** Map
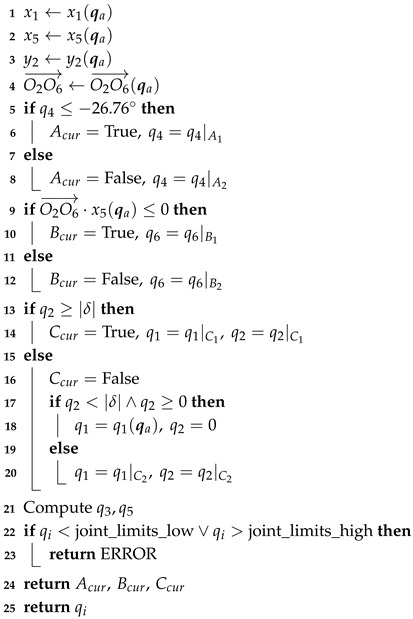


Algorithm 2 describes the exact algorithm for RBRRT. It resembles single-query algorithms such as RRT. A spanning tree (V,E) originating from the initial node $q_{init}$ is connected in the direction of expansion. *V* denotes the set of accessible nodes derived from the sampling technique. *E* is an edge representing the collection of traversable routes established by linking the nodes in *V*. The sampling step size is constant, represented by $d=∥x_{init}-x_{near}∥$. In contrast to RRT, we determine the submanifold of the joint space under constraints on the viable joint nodes before production. Point mapping occurs after the map is identified. Initially, by utilizing the joint values of the preceding node, we may ascertain its distinct submanifold in the joint space under constraints and the precise values of the position and pose in Cartesian space (lines 1–6). Initially, we sample points in Cartesian space to acquire *n* viable points $x_{new}$ within a step size *d* (lines 6–9). Sampling in Cartesian space reduces the complexity of sampling within task limitations, and utilizing the Euclidean paradigm for the pen configuration space simplifies the distance measure. The Map function in Algorithm 1 is employed to find the new sampling points associated with the current $A_{cur}$, $B_{cur}$, and $C_{cur}$ values, allowing determination of whether the current submanifold of the configuration space is under constraints with respect to the previous one; if it is, then the joint space is considered to be in the same conformal state. If consistency is lacking, there is a joint manifold transformation issue akin to the narrow constraint, necessitating an increase in the number of nodes (lines 10–15). Establishing the limited submanifold ensures that the robot manipulator’s orientation remains stable while simultaneously increasing the sample points at variable places, minimizing the computational load. Upon establishing the precise submanifold of the joint space under constraints, the Cartesian-space robot manipulator’s positional orientation and the joint-space joint angle values transform into one-to-one mapping functions with $q_{7}$ as the variable. $q_{7}$ is determined by centering it on the $q_{7}$ of the preceding node and is sampled via a Gaussian distribution, with the maximum step size *d* as the boundary value (lines 16–18).
**Algorithm 2:** RBRRT
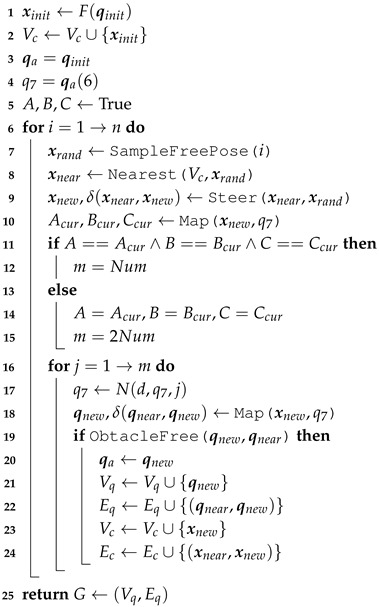


**Figure 5:**
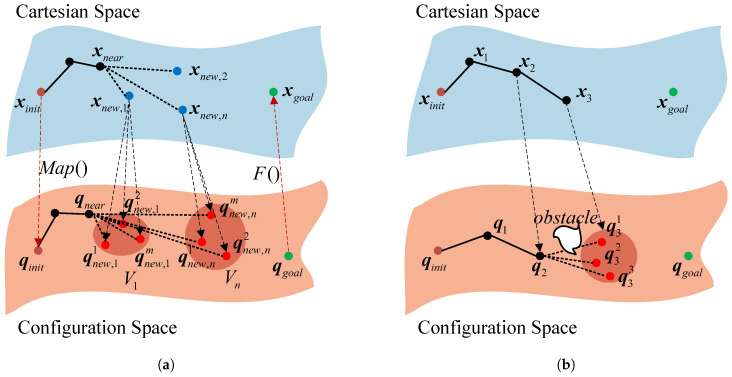
Illustrative diagram of the RBRRT planner. (**a**) Sample point generation strategy for constrained manifolds. (**b**) Connection of sample points on constrained manifolds.

As illustrated in Figure 5a, we sample the Cartesian space to acquire *n* viable nodes and transfer them to the joint space, where each Cartesian-space node corresponds to *m* feasible joint nodes. The *m* nodes represent identical Cartesian-space end-effector positions of the robot manipulator yet have distinct joint space angle values inside the confined manifold of Cartesian space. In contrast to the conventional sampling technique utilizing *n* sampling points, the mapping strategy yields m×n sampling nodes. The nodes are extracted via inverse kinematics parsing, resulting in multiple possible sample locations without substantially increasing the sampling time. If the collision detector progressively determines that $q_{near}$ and $q_{new}$ are free of collisions, then the pair $q_{new}$, ($q_{near}$, $q_{new}$) is incorporated into the joint-space point set $V_{q}$ and edge $E_{q}$. Additionally, the corresponding point $x_{new}$, ($x_{near}$, $x_{new}$) is integrated into the Cartesian-space point set $V_{c}$ and edge $E_{c}$. The graph *G* is produced (lines 19–25). Figure 5b shows that the sampling point $q_{3}^{1}$ of the conventional method on the manifold of the configuration space under constraints is excluded because obstacles obstruct connections to $q_{2}$. In contrast, despite its feasibility, the corresponding Cartesian-space robot manipulator end-effector pose remains included. Our method increases the number of joint nodes associated with the same bit posture, addressing this issue and significantly increasing the graph’s connections.

## 4. Experimental Results

The proposed planner uses the Franka Emika Panda (Franka Robotics GmbH, Munich, Germany) to represent a universal 7-DOF manipulator for simulation and real-world tests under various limitations. In each scenario, the manipulator must reach a designated position and orientation to execute a specific task while remaining within the limits of its joint mobility and circumventing environmental barriers. The machine utilized for motion planning is equipped with an i7 2.6 GHz processor (Lenovo P53, Beijing, China) and 16 GB of RAM. Various traditional algorithms used for comparison are sourced from the open-source Open Motion Planning Library (OMPL) [33]. To assess the efficacy of the proposed planner, all algorithms are executed in Python 3 in the Robot Operating System (ROS) [34] MoveIt! [35]. This section elaborates on scenario building and experimental outcomes in various limited situations.

### 4.1. Comparison with Existing Methods

**Figure 6:**
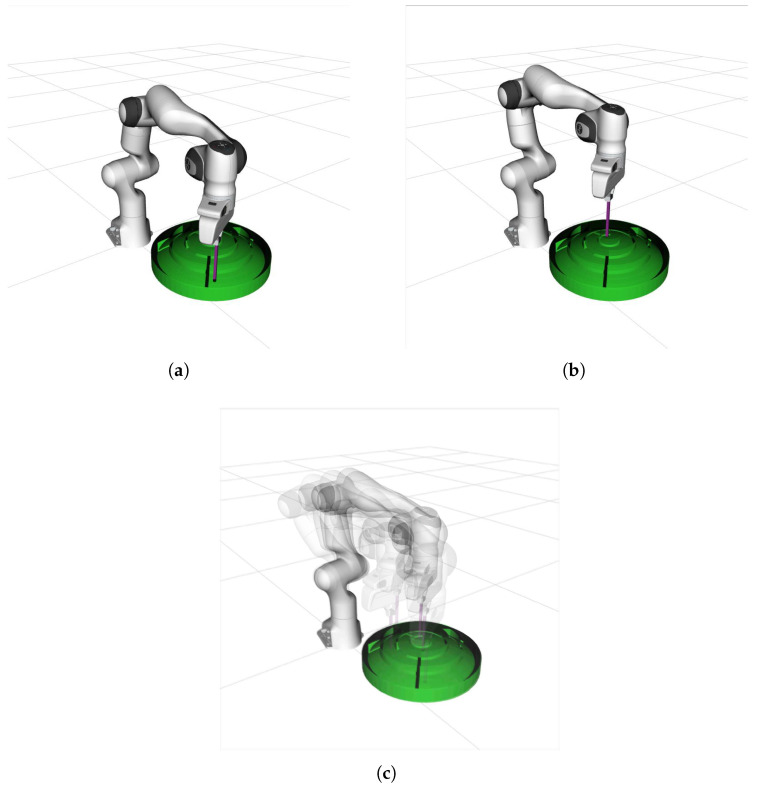
Franka Emika Panda using RBRRT motion planning inside a circular maze. (**a**) Start configuration, (**b**) goal configuration, (**c**) course of motion.

To assess the efficacy of the proposed planner, we devised the scenario depicted in Figure 6. A robot manipulator grasping a slender rod navigates within a curved, circular maze. The maze surface is a sphere centered at (50, 0, −4) with a radius of R=32, and the projection onto the *X*-*O*-*Y* plane is a circle centered at (50, 0) with a radius of r=20. The limitations are measured in centimeters. All the measurements are in centimeters. The restrictions are that the extremity of the slender rod must fit into a slot of dimension 3 in both width and depth created by the spherical surface and that the slender stick has a maximum rotational angle of ε relative to the numerical orientation, encompassing both roll and pitch. The particular restriction $B^{\omega }$ is

$$B^{\omega }=\left[\begin{array}{c} x\in \{x|{\left(x-50\right)}^{2}+y^{2}\leq 20^{2}\}\\ y\in \{x|{\left(x-50\right)}^{2}+y^{2}\leq 20^{2}\}\\ z\in \{x|{\left(x-50\right)}^{2}+y^{2}+{\left(z+4\right)}^{2}\leq 32^{2}\\ \cup {\left(x-50\right)}^{2}+y^{2}+{\left(z+4\right)}^{2}\geq 29^{2}\}\\ \alpha \in [-\varepsilon ,\varepsilon ]\\ \beta \in [-\varepsilon ,\varepsilon ]\\ \gamma \in (-\pi ,\pi ) \end{array}\right]$$

**Figure 7:**
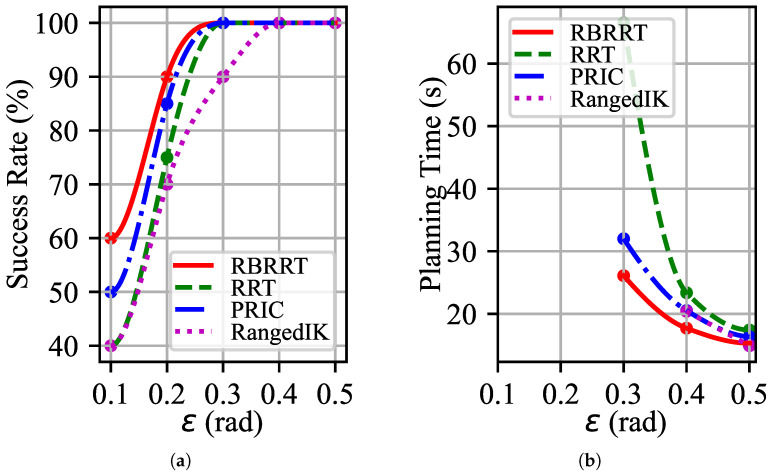
Results of motion planning in a circular maze: (**a**) Success rates of the four planning methods for 20 experiments with different ε values. (**b**) Average planning times of the four planning methods for 20 experiments with different ε values.

The slender stick, secured by the manipulators, is to be moved from the initial location at the designated edge to the target position at the center, adhering to the previously stated limits. The robot manipulator must navigate several constricted tunnels. The proposed redundancy-based planner is compared with the conventional strategy of RRT using traditional projection and the new advanced planners proposed by PRIC (Probabilistic Roadmap with Improved Connectivity) [21] and RangedIK [36] in recent years. PRIC and RangedIK, like the proposed Both PRIC and RangedIK, consider the effect of kinematics on motion planning as the methods proposed in this paper, with the difference that both methods use inverse kinematics with numerical iterations. PRIC is a sampling-based motion-planning method, and RangedIK is an optimization-based motion-planning method. The redundant manipulator reaches the target point and sustains the desired attitude while avoiding obstacles in the surroundings and adhering to work restrictions. The manipulator must comply with the limits of its joints and single configurations to prevent self-collision. All four motion-planning methods were tested 20 times at varying maximum rotation angles in the vertical direction, ε. The success rates and average planning times were recorded, and the experimental findings are illustrated in Figure 7. Notably, in the actual motion-planning task, time is constrained; therefore, we classify the inability to locate the target after 120 s of execution as a failure.

Figure 7a shows the success rates of several motion-planning algorithms. When ε<0.3, none of the strategies can ensure the success of motion planning. Nonetheless, RBRRT has a success rate of approximately 20%, which is superior to that of the most rudimentary RRT when paired with projection. Although PRIC takes into account the effect of kinematics on motion planning, our method is ahead of it mechanistically and still outperforms it by 5% to 10%. Optimization-based motion-planning techniques such as RangedIK can easily fall into minima. Instead, the success rate is relatively low when the constraints are tighter.

Figure 7b shows the average sample durations of various planners. At ε<0.3, we were unable to calculate and fit the average planning time because the success rate was not 100%, but the left-hand side was retained to maintain consistency and correspondence in the graphs. When ε equals 0.3, our technique saves over fifty percent of the time compared with the conventional strategy. However, our planner still has some advantages over more advanced and novel methods. Figure 7 shows that an increased maximum rotation angle ε in the vertical direction, coupled with a relatively flexible constraint, improves success rates and reduces motion times for all motion-planning methods. As the constraint becomes less stringent, the available space for the robot manipulator increases, resulting in comparable planning times across all methodologies. However, when ε is reduced and the limitations are more stringent, the success rates of all planning methods decrease as the planning duration increases. Our proposed planner, RBRRT, achieves a superior success rate and markedly enhanced planning efficiency. In task-oriented planning applications, where a specific success rate is commonly needed, our method can impose more stringent limits while preserving the same success rate.

This contrasts with current techniques that sample within joint-constrained space and project nodes individually. Our planner conducts searches and sampling in Cartesian space, resulting in a substantial reduction in the size and dimensionality of the sampling space. When the vertical direction is rigorously limited, the dimensionality of our sampling space is almost reduced to four dimensions, whereas the conventional projection-based method retains seven dimensions. The Cartesian- and joint-space one-to-many node mapping connections significantly increase the number of viable nodes. This increases node connectivity and the probability of Cartesian space localization. The path cost is reduced within the same timeframe.

### 4.2. Transition Experiments Between Submanifolds

Each submanifold contains a collection of inverse kinematics solutions. Owing to the robot manipulator’s offset structure, there is a large range of motion in a specific direction. Most of the robot manipulator’s tasks can be performed within submanifold I. The value of $q_{4}$ is a crucial criterion in Figure 4, in conjunction with the range of motion of $q_{4}$. The elbow joint’s upward movement is merely 13% of the total range of motion, and in this scenario, the robot manipulator approaches singularity while facing the possibility of self-collision. To examine the general case of the 7-DOF redundant manipulator, we analyze the arm’s transitions between submanifolds under particular conditions.

**Figure 8:**
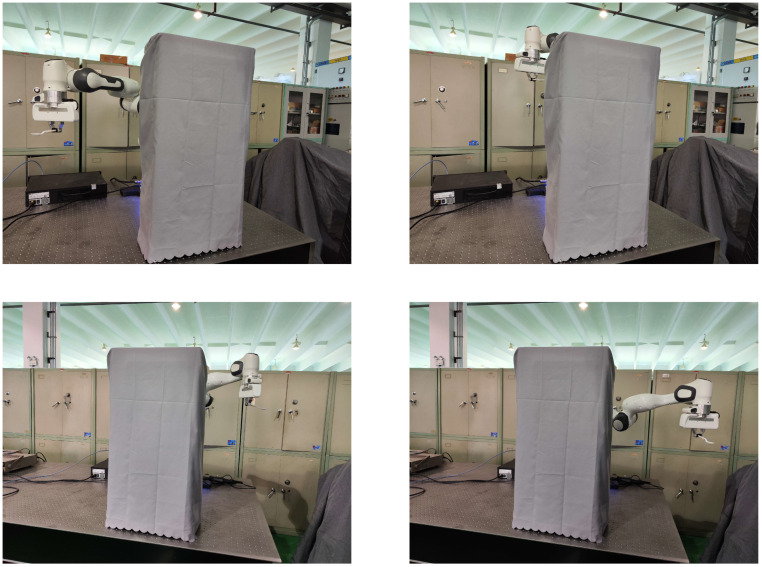
Snapshot of the motion-planning process of the proposed planner for transition experiments between submanifolds.

To test whether the proposed planning method smoothly handles joint space submanifold changes and switching, we conducted an experiment in which the robot manipulator’s terminal end holds a spoon to carry rice past obstacles (Figure 8). The spoon must be horizontal to prevent spilling. The 7-DOF redundant manipulator must consider task constraints and end-effector orientation limits to move from the beginning configuration to the goal pose with the end-effector vertical. We used a laser tracker to calibrate the exact position of fixed spatial obstacles relative to the base of the robot manipulator.

**Figure 9:**
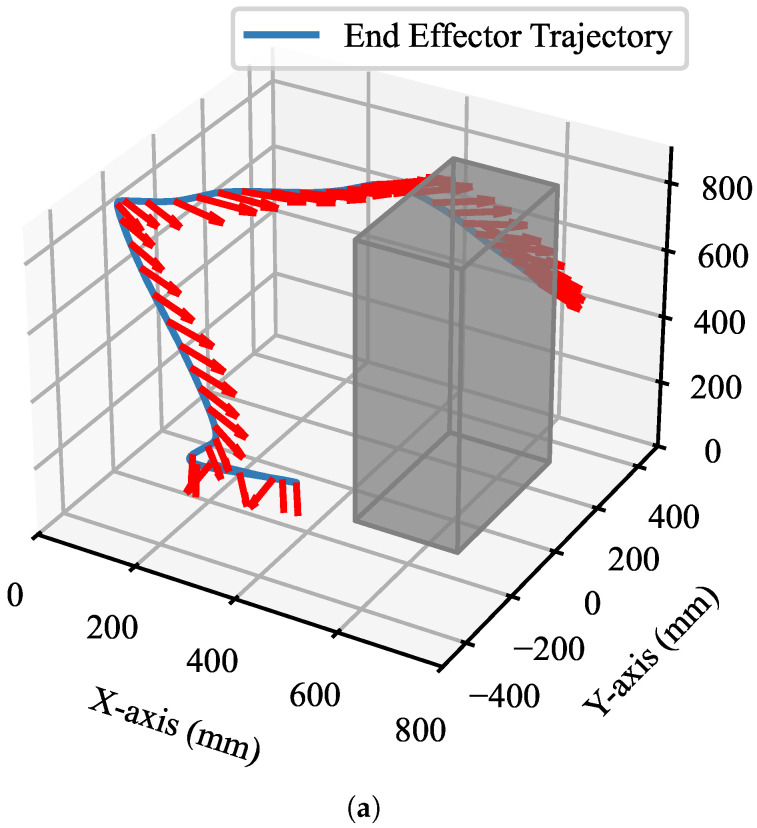

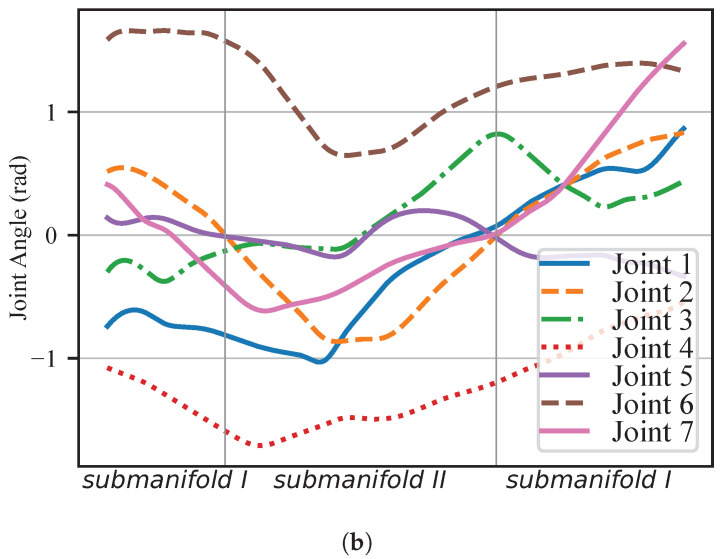
Experiments on submanifold transitions. (**a**) Motion-planning trajectory (blue line) of the end-effector in Cartesian space that satisfies the task constraints, the clamped obstacle bit position (red arrows), and the relative position to the obstacle (gray cube). (**b**) Robot manipulator joint changes that satisfy the task constraints after two submanifold transformations.

Constrained by obstacles and attitude limitations within the workspace, the robot manipulator transforms the joint-space submanifolds twice during its motion. Motion planning in Cartesian space utilizing the proposed planner is illustrated in Figure 9a, where the blue line represents the trajectory of the end-effector in Cartesian space that fulfills the task constraints, the red arrows indicate the held spoons and the gray cubes denote the obstacles within the workspace. The rice remained within the spoon during movement, and the motion in Cartesian space was seamless. The joint transformations of the 7-DOF robot manipulator following two joint submanifold transformations are illustrated in Figure 9b, depicting the joint-space submanifold transitioning from submanifold I to submanifold II and then returning to submanifold I. All joint angle values transition smoothly, with no anomalies observed at the submanifold junction. The experimental results for the end-effector motion trajectories demonstrate that the proposed robot manipulator motion-planning approach transitions smoothly between submanifolds.

## 5. Conclusions

This study proposes an efficient motion-planning approach for the general setup of a 7-DOF robot manipulator in a restricted environment. The constraint-based motion-planning problem is simplified through the utilization of this method, which blends robot manipulator kinematics and motion planning. This is accomplished by sampling and investigating the redundancy of Cartesian space. Using the one-to-many inverse solution of Cartesian-space nodes to joint-space nodes, the method increases the number of viable nodes in joint space, the number of feasible configurations, and the graph connectedness. This is accomplished by increasing the number of options available. Through simulations and testing, it was discovered that the suggested planner is able to plan more quickly and has a higher success rate than the approaches that are based on constraint-based motion planning. It is based on an experiment that looked at task constraints using Franka, a 7-DOF robot manipulator with a general shape, as an example. We studied the theoretical basis for applying a 7-DOF robot manipulator with an approximate configuration to Franka through kinematics because the robot manipulators we tested in the lab are generally biased at the shoulder, elbow, and wrist. This will be expanded upon and applied to other configurations of 7-DOF robot manipulators in later work.

Two concerns will be scrutinized in subsequent study. Human–machine contact and collaboration are becoming increasingly reliant on the utilization of robot manipulators. The proposed motion-planning technique will be integrated with learning methods in order to achieve our goals of enhancing dynamic constraint motion planning and implementing human–computer interaction. It is planned to develop a neural network model for a robot that will be able to carry out demonstration tasks and extract restrictions in order to enable planning without the involvement of humans.

## Data Availability

Data are contained within the article.
